# STIFDB—*Arabidopsis* Stress Responsive Transcription Factor DataBase

**DOI:** 10.1155/2009/583429

**Published:** 2009-10-18

**Authors:** K. Shameer, S. Ambika, Susan Mary Varghese, N. Karaba, M. Udayakumar, R. Sowdhamini

**Affiliations:** ^1^National Centre for Biological Sciences, Tata Institute of Fundamental Research, UAS, GKVK Campus, Bellary Road, Bangalore 560 065, India; ^2^Department of Crop Physiology, UAS, GKVK Campus, Bellary Road, Bangalore 560 065, India

## Abstract

Elucidating the key players of molecular mechanism that mediate the complex stress-responses in plants system is an important step to develop improved variety of stress tolerant crops. Understanding the effects of different types of biotic and abiotic stress is a rapidly emerging domain in the area of plant research to develop better, stress tolerant plants. Information about the transcription factors, transcription factor binding sites, function annotation of proteins coded by genes expressed during abiotic stress (for example: drought, cold, salinity, excess light, abscisic acid, and oxidative stress) response will provide better understanding of this phenomenon. STIFDB is a database of abiotic stress responsive genes and their predicted abiotic transcription factor binding sites in *Arabidopsis thaliana*. We integrated 2269 genes upregulated in different stress related microarray experiments and surveyed their 1000 bp and 100 bp upstream regions and 5′UTR regions using the STIF algorithm and identified putative abiotic stress responsive transcription factor binding sites, which are compiled in the STIFDB database. STIFDB provides extensive information about various stress responsive genes and stress inducible transcription factors of *Arabidopsis thaliana*. STIFDB will be a useful resource for researchers to understand the abiotic stress regulome and transcriptome of this important model plant system.

## 1. Introduction

The challenge of maintaining a balance between a swelling population and the capacity to produce food is increasing day by day. Consequently, food security has become a burning issue for agricultural scientists and economists alike. Increasing crop productivity in view of the escalating population and diminishing cultivable land and natural resources has become vital. However, environmental stresses like drought, salinity, high and low temperatures, high light, and so forth, along with biotic agents like pests and diseases, reduce agricultural yields significantly and consequently affect food security. Developing crops that tolerate environmental stresses, while maintaining productivity, will become a critical requirement for enhancing agriculture in the twenty first century [1]. Understanding the molecular mechanisms that underlie stress tolerance would be the first step in the generation of abiotic stress tolerant crops. To understand plant stress responses, unravelling the mechanisms of regulation of stress responsive genes assumes paramount importance. Gene regulation by Transcription Factors (TFs) is an important facet of stress responsive signal transduction cascades. Transcription factors are regulatory proteins that implement their functions by binding directly to the promoters of target genes in a sequence-specific manner to either activate or repress the transcription of downstream target genes [2]. Transcriptional regulation of genes in response to abiotic stresses like drought, cold, salinity, high light, abscisic acid (ABA), oxidative stress, and so forth, is an emerging area of plant research. As it is impossible to perform biochemical identification and validation of individual genes involved in such complex regulatory events, bioinformatics approaches will be useful to acquire information by integrating diverse data sets and tools. Large scale data integration from multiple experimental and bioinformatics resources will provide a robust platform to understand the major molecular players behind a biological problem. In this paper, we describe the availability of a database designed around stress genes involved in abiotic stress regulation in *Arabidopsis thaliana.* Apart from the database, we also discuss the generic trends of the genes and transcription factors available in STIFDB (Stress responsive TranscrIption Factor DataBase) [3]. STIFDB (available at http://caps.ncbs.res.in/stifdb.) is a database of stress-related genes, which are upregulated in abiotic stress-related microarray experiments. These genes are further analysed to predict all probable abiotic stress responsive Transcription Factor Binding Sites (TFBS) in their regulatory regions, using an efficient, context specific, stress responsive transcription factor binding site prediction algorithm called STIF [4]. We have also integrated Gene Ontology associations [5–7], gene descriptions from TAIR [8, 9], transcription factor-related information from DATF [10], 1000 and 100 base pair up-stream sequences and the 5′UTR sequence for each gene for further analysis, and the stress-signals based stress-profiles to identify the stress responsive impact on individual genes based on different stress signals.

## 2. Materials and Methods

**Figure 1:**
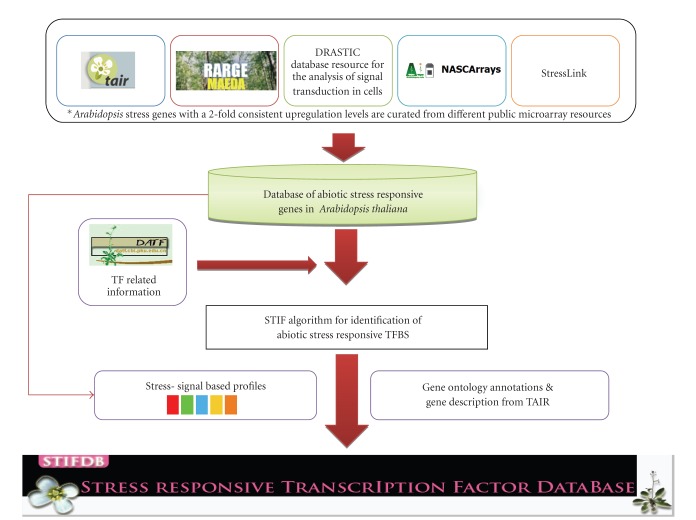
Flow chart of steps involved in the development of STIFDB.

The list of 2269 genes in STIFDB has been compiled from abiotic stress-related microarray experiments. Genes were obtained from gene expression databases like the Nottingham Arabidopsis Stock Centre (NASC) [11], Database Resource for Analysis of Signal Transduction in Cells (DRASTIC) [12], Microarray Expression Data Search of the Riken Arabidopsis Genome Encyclopaedia (RARGE-MAEDA) [13], and the StressLink Database [14]. Genes that are consistently upregulated (upregulated in at least 3 replicates) of microarray experiments in response to various stress treatments like dehydration, drought, osmotic stress, salinity stress, ABA, cold, high light, and oxidative stress across various microarray experiments have been considered as stress responsive and included in the database. In cases where fold increases in expression levels were available, genes with a 4-fold expression change was used to consider the gene as a probable candidate for STIFDB. Sequence segments (1000 bp, 100 bp, and 5′UTR) of genes were obtained from TAIR [9]. The collected sequences were scanned further to identify potential abiotic stress responsive TFBS using the STIF algorithm. In response to abiotic stresses like drought, cold, salinity, high light, heat, salt, and so forth, 10 specific families of transcription factors are known to be involved. 22 HMM-based models [15] of these 10 specific families including subfamilies are used in STIF algorithm to scan for binding sites using STIF algorithm (see Tables 1  and 2). We have also consulted literature to cross-validate the transcription factor binding sites predicted by the STIF algorithm for 29 genes [16]. STIFDB provides the 1000 bp promoter regions, along with their 5′UTR sequences, extracted from TAIR, and identifies known transcription factor binding sites/cis-elements bound by abiotic stress responsive transcription factors. Flow chart of the steps involved in the development of STIFDB is provided in Figure 1.

### 2.1. STIF Algorithm for Prediction of Transcription Factor Binding Sites

STIF [3]—an HMM-based algorithm—is developed to predict transcription factor binding sites in the upstream and 5′UTR regions of genes extracted from TAIR. Statistical significance of the prediction is calculated for each prediction using *Z*-Score and Normalization Score. Program based on STIF method accepts a DNA sequence (Upstream region + 5′UTR) in FASTA format as the input. Extensive experimental results show that abiotic stress responsive transcription factors fall into ten transcription factor families [17, 18]. These are ABI3/VP1, AP2/EREBP, ARF, bHLH, bZIP, HB, HSF, Myb, NAC, and WRKY families, which have a total of 22 subfamilies. Abiotic stress responsive transcription factors largely belong to one of these 22 TF subfamilies (see Tables 1  and 2). Input sequence is scanned using library of these 22 preconstructed stress responsive transcription factor HMMs obtained from literature. Input sequences are scanned for matches to the HMM models. Subsequent to the HMM search, scores of all possible matches in forward and reverse orientations in the upstream regions of stress genes are calculated along with standard deviation and average. Based on STIF search results, hits are scored using significant scoring method. In the final step-Standard deviation, average and significant scores based on hits are used to calculate the *Z*-score and normalization.

### 2.2. STIFDB—Content

STIFDB offers several unique features as well as integrated data from public resources that will be useful for the better understanding of the TFBS and function of the downstream genes.

#### 2.2.1. TFmap

TFmap [19] is a graphical representation of the upstream regions of the stress genes in *Arabidopsis thaliana* with the predicted and the validated transcription factor binding sites marked along with their *Z*-Scores. TFmaps are generated using Bio::Graphics module from Bioperl [20].

#### 2.2.2. TAIR ID

The *Arabidopsis* Information Resource (TAIR) [8, 9] maintains a database of genetic and molecular biology data for the model higher plant *Arabidopsis thaliana*. TAIR ID is used in STIFDB to access the gene-based contents. Users can query the database using TAIR ID.

#### 2.2.3. Gene Ontology

GO annotations [5–7] for the genes in STIFDB are obtained from TAIR. GO annotations will help the users to understand the known functional associations of genes in STIFDB.

#### 2.2.4. Gene Description

Gene description is another feature of functional annotation, that provides a short description of genes along with predicted domain associations from InterPro database [21]. Gene descriptions for genes reported in STIFDB are obtained from TAIR [8, 9].

#### 2.2.5. Gene Names (Including Aliases)

Users can access STIFDB using standard gene names or its aliases reported in TAIR [8, 9] database. For example, TAIR ID-AT4G23600 refers to the single entry in the database with different aliases: CORI3, CORONATINE INDUCED 1, JASMONIC ACID RESPONSIVE 2, and JR2.

#### 2.2.6. Chromosome Position

Chromosome Position refers to the exact location of the given stress gene among the 5 *Arabidopsis thaliana* chromosomes.

#### 2.2.7. References to Publication and Related Resources

References to publications [22] and related resources are provided in each Gene-Related information pages.

#### 2.2.8. Transcription Factor Family Name

This refers to the Transcription Factor Family whose binding site sequence has been located/predicted on a given promoter sequence. This database identifies binding sites of the ten stress responsive transcription factor families and their subfamilies.

#### 2.2.9. Binding Site Information

Binding site refers to the core binding sequence to which a transcription factor binds. The binding site sequences have been characterized in literature reports, and the accompanying references are provided.

#### 2.2.10. Orientation of Binding Sites

Orientation of Binding Sites refers to the DNA strand on which the transcription factor binding site has been located. It can be either on the forward strand or on the reverse DNA strand.

#### 2.2.11. Stress Signals

Stress Signal refers to the type of stress, which, according to literature reports, regulates the transcription factor. Most of the transcription factors dealt with here are regulated by various abiotic stress signals like drought, cold, heat, light, and so forth. A URL is provided to access the database based on different stress signals [23].

#### 2.2.12. *Z*-Score

We have

(1) $$Z\text{-Score}=\frac{\text{Score}-\text{Mean}}{\text{Standard}\;\;\text{Deviation}},$$

where *Z* is the *Z*-Score, Score is the HMM score of the hit, Mean is the Mean of scores of all window slides of query sequence, and the window size depends on the transcription factor binding sites, *Std deviation (Standard Deviation)*—Standard Deviation of mean of all window slides of query sequence.

This algorithm is validated with an experimental data set of 27 stress genes from *Arabidopsis thaliana*. As per that information, we observed that *z*-score for 100 bp and its 5′UTR regions can be seen above 2.0 and for 1000 bp, and its 5′UTR regions can be seen above 1.5.

#### 2.2.13. Normalization Score

We have

(2) $$\text{Normalization}=\frac{𝔎}{𝔅},$$

where *𝔎* is a factor that denotes Top 1st rank of *z*-score of binding site for given TFBS and stress gene/Total number of binding sites for given TFBS and *𝔅* is a factor that denotes Total number of binding sites for all TFBS library and stress gene/Total number of binding sites for all TFBS library and all stress genes. The normalization score explains the distribution of particular TFBS (Transcription Factor Binding Site) in the whole data set of the stress genes. If the normalization numbers are low, then it means that it is well distributed among the data set.

#### 2.2.14. Utilities in STIFDB

**Figure 2:**
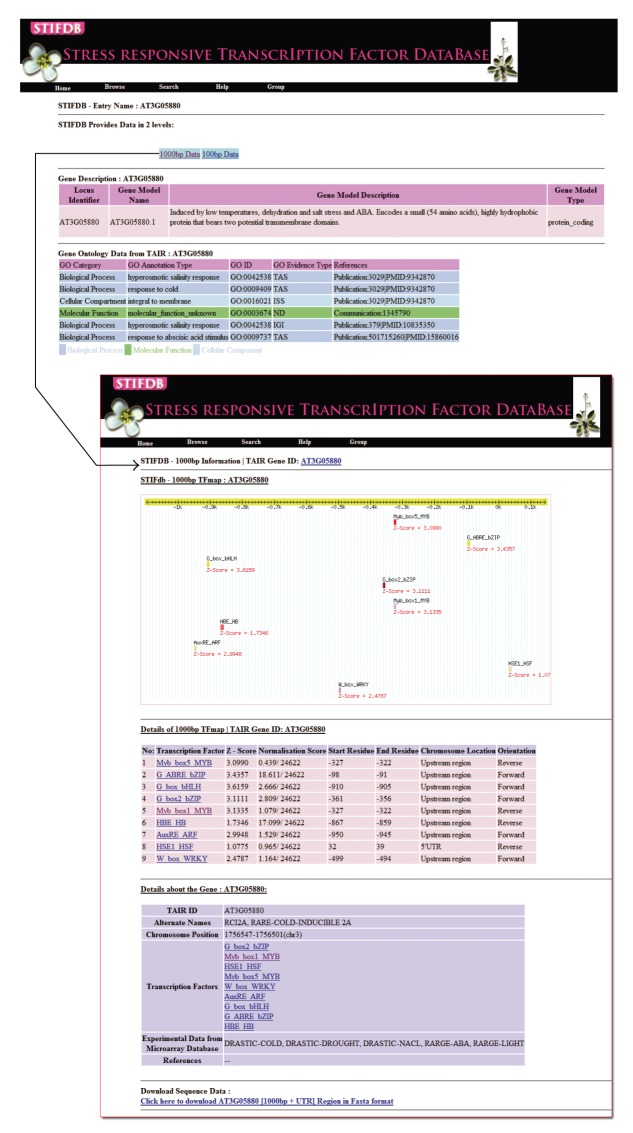
Screenshot of STIFDB.

STIFDB is organised such that the users can browse using four criteria like chromosome number, transcription factors, stress signal profiles, and sorted list of TAIR locus IDs. Users can search the STIFDB using TAIR locus IDs, Gene alias names, and stress signals. A BLAST- (blastn-) [24] based search tool is also implemented to search the database of 1000 bp promoter sequences of 2629 genes in STIFDB. A detailed screenshot of STIFDB with various features are provided in Figure 2.

#### 2.2.15. Technical Details

STIFDB is developed on a MySQL [25] backend. Web interface of STIFDB is developed using HTML and JavaScript. Perl-CGI programs are used for the development of search, query, and retrieval system. All scripts for parsing, searching, statistics, and other calculations are coded for the STIF algorithm in Perl [26]. The source code of STIF program is available from the corresponding author upon request.

## 3. Results and Discussion

**Figure 3:**
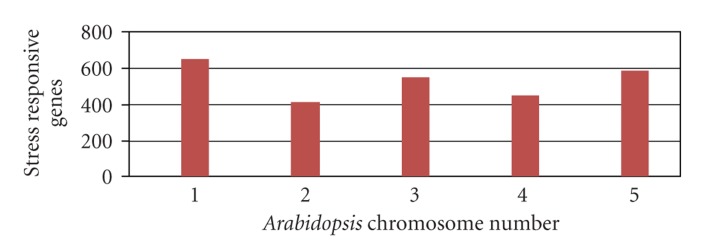
Chromosomewise distribution of abiotic stress responsive genes in STIFDB.

**Figure 4:**
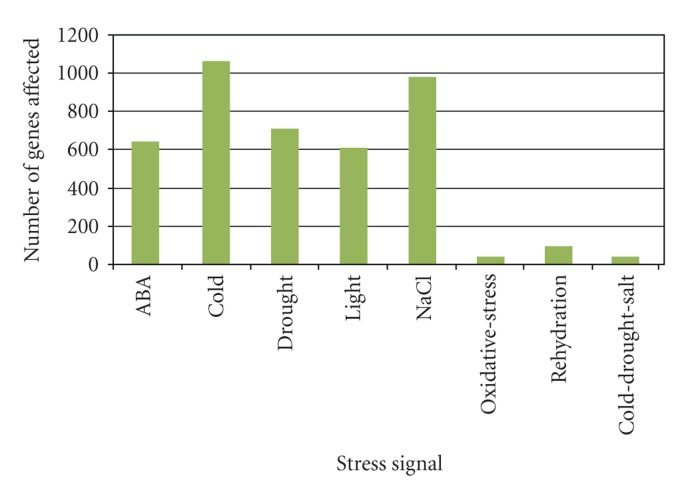
Distribution of individual stress signal that affects genes in STIFDB.

**Figure 5:**
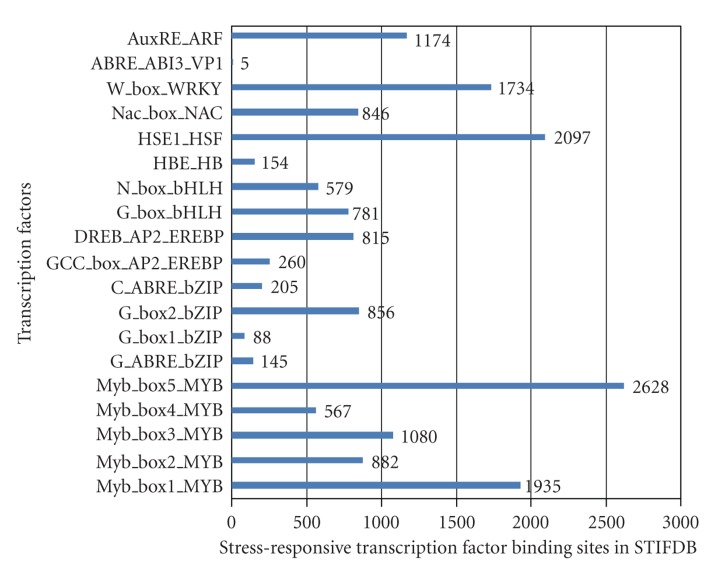
Frequency of transcription factor binding sites in STIFDB.

With a growing world population, food security is a high concern as cultivation of food crops are in risk due to various biotic and abiotic stress factors. Better understanding of plant stress response mechanisms and application of knowledge derived from integrated experimental and bioinformatics approaches are gaining importance. Abiotic stresses cause up to 30% yield losses [27], and hence an explicit data organisation and a clearer understanding of the regulation of abiotic stress responsive genes have become crucial. With genomic sequence data available, bioinformatics tools have been valuable for large scale analyses of genes [2] and understanding gene regulation. STIFDB is a database of abiotic stress responsive genes, identified as responsive to various abiotic stress signals based on publicly available, genome wide stress microarray data. STIFDB is a useful resource to analyse the promoters of these abiotic stress responsive genes for potential stress-specific transcription factor binding sites, which would provide insights into the regulation of these stress responsive genes by upstream transcription factors. It also provides clues towards the stress signal that affects the transcription of this gene, which might offer clarity about signal specific regulation of these genes. List of genes in STIFDB indicates that abiotic stress responsive genes seem to be roughly the same numbers on all chromosomes. Chromosome-wise distribution of abiotic stress responsive genes in STIFDB is provided in Figure 3. Distribution of genes responsive to specific abiotic stress signals indicates that numerous genes are regulated in response to cold, drought, salinity, light and external ABA, and a lesser subset of genes that respond to oxidative stress and rehydration. Distribution of individual stress signal that affects genes in STIFDB is provided in Figure 4. There are also 41 genes that are expressed in response to multiple abiotic signals, cold, drought, and salinity. Analysing these genes as subsets or individually, would offer clues to understanding the individual stress transciptomes better, and analysing the promoters of these genes could provide insights into the regulation of these genes in response to their specific stress signal. We have further analysed the number of TFBS on the promoters of these abiotic stress responsive genes and have identified varying numbers of stress-specific TFBS. This gives a broad indication that the genes in STIFDB are indeed abiotic stress responsive. There seems to be greater numbers of certain TFBS than others. This could partly be due to the differences in the length of these cis-elements. Frequency of individual transcription factor binding sites on 2629 genes in STIFDB is provided in Figure 5. STIFDB would be a very useful tool to understand abiotic transcriptome and the regulatory events of abiotic stress genes in *Arabidopsis*.

## 4. Conclusion

Experimental validation and evidence about how many of these TFBS actually bind a TF to bring about regulation of their downstream gene *in vivo* is still lacking suggesting that we need to be cautious about the hits and the seeming false positives. It also needs to be determined if a greater number of stress-specific TFBS on the promoter, a particular gene, means a greater role of that particular TF in its regulation. It is also would be worthwhile to analyse the promoters of subsets of genes that are regulated by specific stresses, to identify patterns of TFBS, which would have potential roles in the regulation of downstream genes responsive to a particular stress. Therefore, STIFDB provides a platform to understand the stress-regulome of abiotic stress responsive genes in plants. STIFDB will be a highly useful resource for a researcher working on abiotic stress responses in plants.

## Figures and Tables

**Table 1:** Table of transcription factors considered.

No.	Family name	Subfamily	Stress signal	Reference (Stress signal)	Name of the Cis-element	Cis-element	Reference (Cis-element)
1	ABI3/VP1		ABA	Plant J. 2000; 24(1): 57–66	distB ABRE	GCCACTTGTC	Plant J. 2000; 24(1): 57–66

2	AP2/EREBP	EREBP-ERF	Cold, Drought	The Plant Cell, 1998; 10: 1391–1406.	GCC-box	GCCGCC	The Plant Cell, 1998; 10: 1391–1406.
		DREB	Cold, Drought	Proc. Natl. Acad. Sci., 1997; 94: 1035–1040	CRT/DRE	(A/G)CCGAC	Proc. Natl. Acad. Sci., 1997, 94: 1035–1040

3	ARF		Auxin	PNAS, 1999; 96(10): 5844-9	AuxREs	TGTCTC	PNAS, 1999; 96(10): 5844-9

4	BHLH/myc		NaCl, ABA, Drought	The Plant Cell, 2003; 15: 63–78	N box	CACG(G/A)C	The Plant Cell, 2003; 15: 63–78
					G box	CACGTG	The Plant Cell, 2003; 15: 1749–1770

5	bZIP		ABA, Drought	Current Opinion in Plant Biology 2000; 3: 217–223	G box1	CCACGTGG	The Plant Cell, 1992; 4: 1309–1319
					G box2	TGACG(T/C)	The Plant Cell, 1992; 4: 1309–1319
					G/ABRE	(C/T)ACGTGGC	Journal Of Biological Chemistry, 2000; 275(3): 1723–1730
					C/ABRE	CGCGTG	Journal Of Biological Chemistry, 2000; 275(3): 1723–1730

6	HB		ABA, Drought	*Plant Molecular Biology,* 1998; 37: 377–384.		CAATNATTG	Nat. Struct Biol, 1999; 6: 464–470

7	HSF		Drought, Cold, Heavy-metal stress and oxidative stress	Plant Physiol. 1998; 117: 1135–1141	HSE	TTCNNGAAGAANNTTC	Nat. Struct Biol, 1999; 6: 464–470

8	MYB		Dehydration, Wounding	The Plant Cell, 1993; 5: 1529–1539		(T/C)AAC(G/T) G	*Genes & Dev.*;1990; 4: 2235–2241
						CC(T/A)ACC	Genetics, 1998; 149: 479–490.
						TAACTG	Plant Journal, 1996; 10(6): 1145–1148
						CC(TA)AACC	Genetics, 1998; 149: 479–490.
						(C/T)AACN(A/G)	The Plant Journal, 2003; 33: 259–270

9	NAC		Drought, high salinity and ABA	The Plant Cell, 2004; 16: 2481–2498.		CATGTG	Plant Mol Biol.; 2002; 50(2): 237–48.

10	WRKY		Biotic stress (pathogen attack)	*Plant Physiology*, 2002, 129: 661–677	W box	(T)TGAC(C/T)	*Plant Molecular Biology*; 51: 21–37, 2003.
			Abiotic stress (wind, rain, hail)				

**Table 2:** Details of transcription factors and subfamily members available in STIFDB.

No.	Transcription factor family	Subfamily members
1	MYB	Myb_box1, Myb_box2, Myb_box3,Myb_box4, Myb_box5
2	bZIP	C_ABRE, G_ABRE, G_box1, G_box2
3	EREBP	DREB_AP2, GCC_box_AP2
4	bHLH	G_box, N_box
5	VP1	ABRE_ABI3
6	ARF	AuxRE
7	WRKY	W_box
8	NAC	Nac_box
9	HB	HBE
10	HSF	HSE1
